# Recurrent Tubo-Ovarian Abscesses in a Non-sexually Active Adolescent: A Case Report and Review of Atypical Risk Factors

**DOI:** 10.7759/cureus.84671

**Published:** 2025-05-23

**Authors:** Samuel Sabzanov, Marc Ganz, Betsalel Adout, Neekoo Farahmandpour, Jonathan Colarusso, Daniel Yusupov, Nekhama Riznyk, Benjamin Mishail, Daniel Miller

**Affiliations:** 1 Internal Medicine, State University of New York Downstate Health Sciences University, Brooklyn, USA; 2 Urology, Maimonides Medical Center, Brooklyn, USA; 3 Internal Medicine, University Hospital of Brooklyn, Brooklyn, USA; 4 Internal Medicine, Icahn School of Medicine at Mount Sinai, New York City Health + Hospitals/Queens, New York, USA

**Keywords:** pelvic inflammatory disease, pid, toa, tubo-ovarian abscess, urology

## Abstract

Tubo-ovarian abscesses (TOAs) are serious infections that typically arise in the context of pelvic infections. While they are most often linked to specific risk factors, their occurrence in non-sexually active individuals is uncommon and presents unique diagnostic and management challenges. This case describes a 14-year-old adolescent with recurrent TOAs. She presented with abdominal pain, fever, and laboratory findings suggestive of infection. Imaging confirmed the presence of a pelvic abscess, requiring a combination of antibiotics and procedural intervention. A thorough evaluation did not reveal any clear predisposing factors. Her condition improved with treatment, and follow-up care was arranged to monitor for recurrence. This case highlights the importance of considering TOA in the differential diagnosis of abdominal pain, even in patients without commonly associated risk factors. Early recognition and a multidisciplinary approach are essential for effective management and improved outcomes.

## Introduction

Tubo-ovarian abscesses (TOAs) are severe complications of pelvic inflammatory disease, which is classically associated with sexually transmitted infections such as *Chlamydia trachomatis* and *Neisseria gonorrhoeae* [1]. The condition predominantly occurs in sexually active individuals, with the pathogenesis closely linked to ascending infections from the lower genital tract. However, the occurrence of TOAs in non-sexually active (NSA) females is exceedingly rare, representing a diagnostic and clinical challenge due to its atypical presentation and the absence of traditional risk factors [2,3]. This subset of patients often presents with vague symptoms such as abdominal pain and fever, which can mimic other, more common conditions such as appendicitis or urinary tract infections [4-6]. As a result, delayed recognition and treatment are not uncommon, potentially leading to life-threatening complications such as rupture, peritonitis, or sepsis [6].

The pathogenesis of TOAs in NSA females is not well understood but has been linked to several non-traditional risk factors, including obesity, recurrent urinary tract infections, poor hygiene [7,8], and congenital anomalies of the genitourinary tract [9]. Case series and reports have also implicated conditions such as obstructed hemivaginal ipsilateral renal anomalies, Crohn’s disease, or prior pelvic surgeries as potential contributors [10]. Moreover, the diagnostic process can be further complicated by overlapping clinical features with more common etiologies of pelvic or abdominal pain in adolescents [2]. The management of TOAs in NSA females requires a high degree of clinical suspicion, with prompt imaging, multidisciplinary involvement, and timely intervention to prevent severe morbidity or mortality.

This case report describes a recurrent TOA in a 14-year-old non-sexually active adolescent female, emphasizing the diagnostic and therapeutic challenges of this unusual presentation. The patient’s prior history of recurrent adnexal abscesses requiring drainage, along with atypical risk factors, highlights the importance of considering TOA even in the absence of sexual activity. By providing a detailed account of the diagnostic process, management strategy, and follow-up, this case aims to raise awareness of this rare condition and highlight the need for a broader differential diagnosis in NSA females presenting with abdominal or pelvic pain. Ultimately, increasing recognition of TOA in this population has important implications for reducing diagnostic delays, optimizing treatment, and improving outcomes in an understudied and vulnerable patient group.

## Case presentation

A 14-year-old African American obese female presented to the emergency department with a five-day history of right-sided abdominal pain accompanied by loose stools and non-bilious, non-bloody vomiting. Her mother reported that the patient had experienced two prior episodes of ovarian cysts requiring drainage. Both episodes involved prolonged hospital stays lasting two to three weeks and required interventional management. The patient denied any sexual activity, abnormal vaginal discharge, or urinary symptoms. Her menstrual history was unremarkable, with regular cycles, and her last menstrual period occurred three weeks before presentation.

On evaluation, the patient appeared febrile, with a temperature of 38.4°C (101.1°F), and tachycardic, with a heart rate of 129 beats per minute. Her blood pressure was 104/68 mmHg, her respiratory rate was 20 breaths per minute, and her oxygen saturation was 98%. She was obese, with a body mass index greater than 34 kg/m². Abdominal examination revealed tenderness in the right and left lower quadrants, as well as a firm, fixed, tender palpable mass in the periumbilical region. There was no guarding or rebound tenderness.

Laboratory evaluation revealed leukocytosis with a white blood cell count of 14,200/μL and an absolute neutrophil count of 9.4 × 10³/μL. Inflammatory markers were elevated, including a C-reactive protein level of greater than 80 mg/L. Liver enzymes were marginally elevated, with an alanine aminotransferase of 95 U/L and an aspartate aminotransferase of 125 U/L. Tumor markers were negative except for an elevated cancer antigen-125 (CA-125), which was considered non-specific. A urinalysis suggested a urinary tract infection, and a urine culture subsequently grew *Escherichia coli*. A sexually transmitted infection panel was negative (Table 1).

**Table 1:** Laboratory findings. CBC: complete blood count; ANC: absolute neutrophil count; BUN: blood urea nitrogen; CRP: C-reactive protein; ALT: alanine aminotransferase; AST: aspartate aminotransferase; CA-125: cancer antigen-125; AFP: alpha-fetoprotein; beta-hCG: beta-human chorionic gonadotropin; LDH: lactate dehydrogenase; WBCs: white blood cells; RBCs: red blood cells; STI: sexually transmitted infection; NAAT: nucleic acid amplification test; RPR: rapid plasma reagin; NK: natural killer

Laboratory test	Result	Reference range
CBC with differential		
WBC	14.2 × 10³/μL	4.5–11.0 × 10³/μL
Hemoglobin	11.4 g/dL	12.0–16.0 g/dL
Hematocrit	35.2%	36–46%
Platelets	390 × 10³/μL	150–450 × 10³/μL
ANC	9.4 × 10³/μL	1.5–8.0 × 10³/μL
Lymphocytes	2.1 × 10³/μL	1.0–3.5 × 10³/μL
Basic metabolic panel		
Glucose	100 mg/dL	70–110 mg/dL
BUN	7 mg/dL	8–21 mg/dL *(Low)*
Creatinine	0.7 mg/dL	0.4–0.8 mg/dL
Sodium	135 mEq/L	136–145 mEq/L (Low)
Potassium	5.2 mEq/L	3.5–5.1 mEq/L (High)
Chloride	95 mEq/L	98–107 mEq/L (Low)
CO₂ (bicarbonate)	27 mEq/L	21–31 mEq/L
Calcium	8.9 mg/dL	8.6–10.3 mg/dL
Inflammatory markers		
CRP	87 mg/L	<5 mg/L
Liver function tests		
ALT	95 U/L	7–56 U/L
AST	125 U/L	10–40 U/L
Tumor markers		
CA-125	53 U/mL	<35 U/mL
AFP	2.1 ng/mL	<10 ng/mL
Beta-hCG (quantitative)	<5.0 mIU/mL	<5.0 mIU/mL
LDH	187 U/L	105–333 U/L
Urinalysis		
Appearance	Cloudy	Clear
Color	Yellow	Yellow
Specific gravity	1.020	1.005–1.030
Leukocyte esterase	Positive	Negative
Nitrites	Positive	Negative
WBCs	20–50 /hpf	0–5 /hpf
RBCs	0–2 /hpf	0–2 /hpf
Protein	Negative	Negative
Glucose	Negative	Negative
Urine culture	*Escherichia coli* >100,000 CFU/mL	No growth
STI panel (NAATs)		
Chlamydia trachomatis	Not detected	Not detected
Neisseria gonorrhoeae	Not detected	Not detected
Trichomonas vaginalis	Not detected	Not detected
HIV antigen/antibody	Non-reactive	Non-reactive
RPR (syphilis screening)	Non-reactive	Non-reactive
Immunoglobulin panel		
Immunoglobulin G (IgG)	973 mg/dL	700–1,600 mg/dL
Immunoglobulin A (IgA)	129 mg/dL	70–400 mg/dL
Immunoglobulin M (IgM)	112 mg/dL	40–230 mg/dL
HIV antigen/antibody (fourth generation)	Non-reactive	Negative
Lymphocyte subset panel		
CD3+ T cells	1,480 cells/μL	690–2,540 cells/μL
CD4+ T-helper cells	850 cells/μL	410–1,590 cells/μL
CD8+ T-cytotoxic cells	510 cells/μL	190–1,140 cells/μL
CD19+ B cells	280 cells/μL	100–610 cells/μL
CD16+/CD56+ NK cells	210 cells/μL	90–600 cells/μL
Inflammatory bowel disease markers		
Fecal calprotectin	38 µg/g	<50 µg/g
CRP (repeat, non-acute)	3.1 mg/L	<5 mg/L

**Figure 1:**
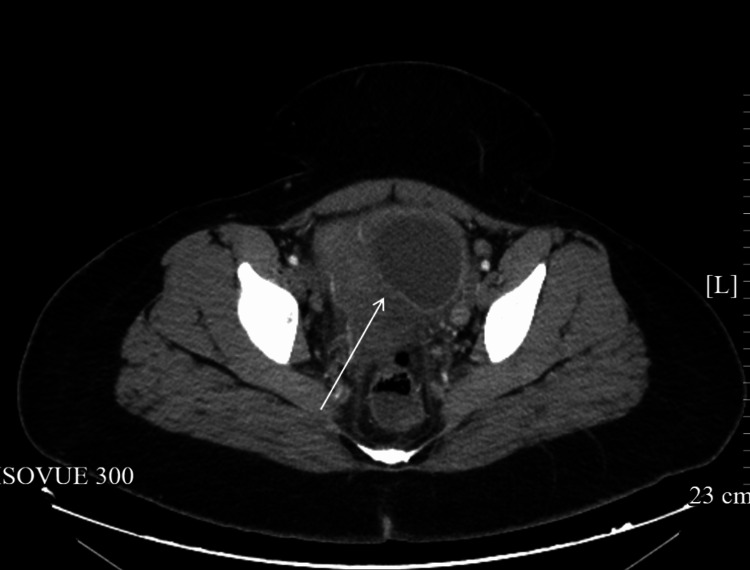
CT with contrast revealing a large cyst consistent with a tubo-ovarian abscess. The arrow points toward the large cyst.

Initial CT scan with contrast of the abdomen and pelvis revealed a large, multilobulated pelvic mass measuring 14 × 9 cm, with the largest cystic structure measuring 10.5 × 7.2 cm. The uterus was compressed and shifted rightward. No evidence of appendicitis was noted (Figure 1).

The findings were consistent with a TOA. Based on these findings, the patient was admitted to the pediatric floor for further management. Broad-spectrum intravenous antibiotics, including cefoxitin, doxycycline, and metronidazole, were initiated. Obstetrics and gynecology were consulted, and interventional radiology was brought in to plan for potential abscess drainage.

**Figure 2:**
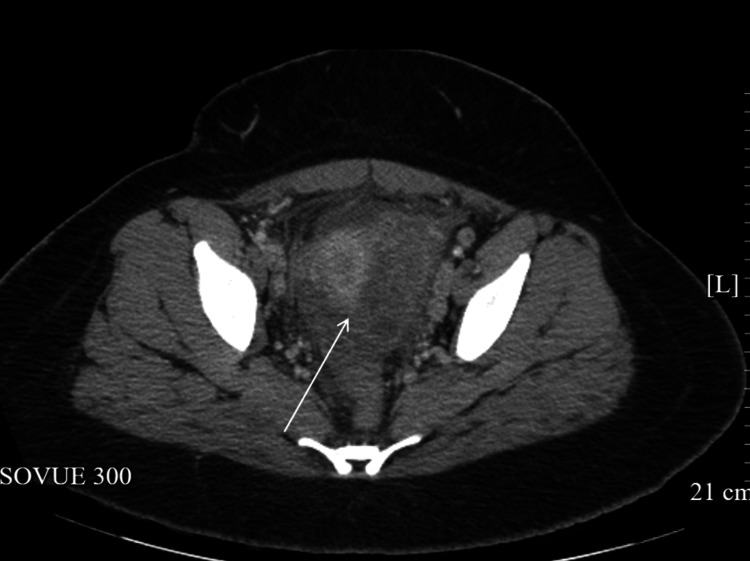
Follow-up CT of the abscess status post-drainage. The arrow points toward the resolving abscess.

On the third day of hospitalization, the patient underwent ultrasound-guided drainage of the right lower quadrant abscess, which yielded 500 mL of purulent material, which was sent for gram stain and culture. A 12-French pigtail catheter was successfully placed to facilitate continued drainage. Subsequent imaging demonstrated a marked reduction in the size of the dominant pelvic mass, which decreased from 14 × 9 cm to 3-4 cm (Figure 2).

Residual loculated fluid collections were observed, along with generalized pelvic edema. New findings of reactive retroperitoneal lymphadenopathy, measuring 1.5-2.5 cm, were also noted. Follow-up pelvic ultrasound revealed a tubular cystic structure in the left adnexa measuring 5.84 × 1.85 × 3.63 cm, consistent with hydrosalpinx, as well as residual fluid collections. No significant free fluid was observed.

A comprehensive workup was performed to investigate potential underlying causes of the recurrent TOAs. Tests for immunodeficiency and Crohn’s disease were negative. Evaluation for congenital genitourinary anomalies did not reveal any abnormalities. The patient’s symptoms improved significantly following abscess drainage and continued antibiotic therapy. By the time of discharge, inflammatory markers had decreased, and imaging showed near-complete resolution of the abscess. At this point, the catheter was removed. The Gram stain revealed Gram-positive cocci in clusters, but the culture grew more than four bacteria, and there were concerns for contamination. The patient was discharged on a 14-day course of oral doxycycline and metronidazole and advised to follow up with gynecology for long-term monitoring and further evaluation to prevent recurrence.

## Discussion

TOAs are most commonly associated with sexually transmitted pathogens ascending from the lower genital tract, making their occurrence in NSA females extremely rare and often overlooked during the diagnostic process [1,6]. In NSA patients, the absence of sexual activity leads to a lower index of suspicion for TOA, frequently resulting in delays in diagnosis and treatment, as illustrated in this case [6]. Despite recurrent presentations, the patient’s condition was initially attributed to ovarian cysts, underscoring the diagnostic challenge when TOA presents without classical risk factors.

Several studies have reported that NSA adolescents who develop TOA often have underlying conditions or risk factors that differ from the general population. These include obesity, recurrent urinary tract infections, poor perineal hygiene, congenital genitourinary anomalies, or gastrointestinal disorders such as Crohn’s disease [2,11]. However, in many cases, including the one described here, no clear predisposing factor can be identified, suggesting a possible multifactorial or idiopathic etiology in this subset of patients [12].

Imaging plays a critical role in the evaluation of pelvic masses in adolescents. Ultrasound is typically the first-line modality, though CT or MRI may be required for better anatomical resolution or to rule out differential diagnoses such as appendicitis, neoplasm, or ovarian torsion [13]. In this case, CT revealed a large, complex pelvic mass with features classic for TOA, prompting further intervention. The use of image-guided percutaneous drainage in conjunction with broad-spectrum antibiotics has been shown to be both effective and fertility-preserving in adolescent patients [14]. While surgical intervention may be necessary in cases of rupture or non-responsiveness, a conservative approach is preferred when feasible.

Notably, this patient experienced recurrent TOA despite appropriate treatment and resolution on prior admissions. Recurrent TOAs are uncommon and may point to anatomical, immunological, or microbiological factors that predispose to reinfection [15]. While evaluations for Crohn’s disease, congenital genitourinary anomalies, and immunodeficiencies were negative in this case, the possibility of subtle or yet unidentified factors remains. Elevated CA-125, although nonspecific, further supports the inflammatory burden typically associated with TOA rather than malignancy in this context [16].

The microbiological findings of *E. coli* in the urine culture raise the possibility of an ascending urinary tract infection acting as a nidus for pelvic infection, particularly in obese adolescents, where perineal hygiene may be more difficult to maintain. Prior studies have found *E. coli* and other enteric organisms in TOA cultures of NSA patients, differing from the polymicrobial flora seen in sexually active individuals [17-19].

Ultimately, the case reinforces the importance of maintaining a broad differential diagnosis when evaluating pelvic masses in adolescent females, regardless of reported sexual history. Clinicians should remain aware of the rare but real possibility of TOA in NSA patients, especially when imaging findings and clinical symptoms align. Prompt diagnosis and intervention can prevent serious complications, such as abscess rupture, sepsis, or future infertility.

## Conclusions

This case highlights the diagnostic challenges and management considerations for TOAs in NSA adolescent females. The presentation of abdominal pain, fever, and leukocytosis can mimic other conditions, such as appendicitis or complicated urinary tract infections, which may delay the diagnosis. In this case, the patient’s prior history of recurrent adnexal masses requiring drainage suggested an underlying predisposition, though no specific etiology was identified. Prompt multidisciplinary intervention, including antibiotic therapy and ultrasound-guided drainage, was essential in preventing severe complications such as abscess rupture and peritonitis. This case underscores the importance of considering TOA in the differential diagnosis of pelvic masses in NSA females and highlights the need for thorough follow-up to address potential risk factors and prevent recurrence.
